# Effects of Relaxing and Arousing Music during Imagery Training on Dart-Throwing Performance, Physiological Arousal Indices, and Competitive State Anxiety

**DOI:** 10.3389/fpsyg.2018.00014

**Published:** 2018-02-05

**Authors:** Garry Kuan, Tony Morris, Yee Cheng Kueh, Peter C. Terry

**Affiliations:** ^1^Exercise and Sports Science Programme, School of Health Sciences, Universiti Sains Malaysia, Penang, Malaysia; ^2^College of Sport and Exercise Science, Institute of Sport, Exercise and Active Living, Victoria University, Melbourne, VIC, Australia; ^3^Unit of Biostatistics and Research Methodology, School of Medical Sciences, Universiti Sains Malaysia, Penang, Malaysia; ^4^Division of Research and Innovation, University of Southern Queensland, Toowoomba, QLD, Australia

**Keywords:** physiological arousal, relaxing music, arousing music, imagery, fine-motor skill performance

## Abstract

Music that is carefully selected to match the requirements of activities and the characteristics of individuals has been shown to produce significant impacts on performance enhancement (Priest et al., 2004). There is also evidence that music can enhance imagery (Grocke and Wigram, 2007), although few studies have investigated the effects of music on imagery in the context of sport skills. In the present study, the effects of relaxing and arousing music during imagery on dart-throwing performance, physiological arousal indices, and competitive state anxiety, were investigated among 63 novice dart throwers. Participants had moderate-to-high imagery ability and were randomly assigned to unfamiliar relaxing music (URM), unfamiliar arousing music (UAM), or no music (NM) groups. Performance was assessed by 40 dart throws at a concentric circles dartboard before and after 12 imagery sessions over 4 weeks. Measures of galvanic skin response (GSR), peripheral temperature (PT), and heart rate (HR) were taken during imagery sessions 1 and 12, and the Competitive State Anxiety Inventory-2 Revised (CSAI-2R) was administered prior to the pre- and post-intervention performance task. Dart-throwing gain scores were significantly higher for URM than for UAM and NM, with no significant difference between UAM and NM (URM = 37.24 ± 5.66, UAM = 17.57 ± 5.30, and NM = 13.19 ± 6.14, *F*_2,62_ = 5.03, *p* = 0.01, η^2^ = 0.14). GSR, PT, and HR reflected lower arousal for URM than for UAM or NM. Significant decreases in somatic anxiety were evident for URM and UAM but not NM. Significant decreases in cognitive anxiety were evident for URM and NM but not UAM. Significant increases in self-confidence were evident for URM but not UAM or NM. Performance improved in all three conditions but URM was associated with the largest performance gain, the lowest physiological indices of arousal, and the most positive CSAI-2R profiles. Listening to relaxing music during imagery may have benefits for performance in other fine motor skills.

## Introduction

Imagery techniques have been acclaimed as a “central pillar of applied sport psychology” and are included in almost all psychological skills training programs for athletes (Perry and Morris, 1995, p. 339). Imagery has been defined as “the creation or re-creation of an experience generated from memorial information, involving quasi-sensorial, quasi-perceptual and quasi-affective characteristics, that is under the volitional control of the imager, and which may occur in the absence of the real stimulus antecedents normally associated with the actual experience” (Morris et al., 2005, p. 19).

Athletes report using imagery techniques for varied purposes, including learning and practicing skills, during pre-performance routines and game planning strategies, for previews and reviews of performance, for mental warm-ups and the development of psychological skills, for problem solving and stress management, for increasing concentration and confidence levels, and during injury rehabilitation (Morris et al., 2005; Strachan and Munroe-Chandler, 2006). Researchers have investigated several different aspects of imagery use in sport, including imagery objectives, types of imagery, measurement of imagery ability, psychophysiological factors, and performance enhancement imagery (Collins and Hale, 1997; Hall et al., 1998; Hall, 2001; Watt et al., 2004). Despite these extensive research efforts, understanding optimal imagery use in sport remains a challenge, requiring further investigations aimed at deriving greater benefit from imagery training. This represents a gap in imagery research that is yet to be examined comprehensively.

Music plays an influential role in many sports, being integral to performance in gymnastics, synchronized swimming, and ice dancing, or is used for motivational and entertainment purposes, to elicit patriotism and pride, and enhance the psychological state and performance of athletes (Terry and Karageorghis, 2011). Demonstrated benefits of music in sport and exercise include arousal control, enhanced affective states, lowered perceived exertion, improved performance, and greater physiological efficiency (Boutcher and Trenske, 1990; Terry et al., 2012; Bood et al., 2013; Hutchinson and Karageorghis, 2013). Music can clearly influence perceived relaxation and excitation levels, and its influence on physiological indicators, such as galvanic skin response (GSR), peripheral temperature (PT), and heart rate (HR), is also well established (Burns et al., 1999; Lundqvist et al., 2009; Salimpoor et al., 2009, 2011; Kuan et al., 2017).

Very few studies have investigated use of imagery accompanied by music as a way to enhance sports performance. Karageorghis and Lee (2001) compared the effects of motivational music, imagery, and a combination of both on an isometric muscular endurance task that required participants to maintain dumbbells in a cruciform position. The combination of music and imagery yielded greater endurance than music or imagery alone, although it was unclear if music and imagery interacted in some way to produce an ergogenic effect, or if enhanced endurance was due to the summation of the motivational impact of the music and the motivational impact of the imagery. Pain et al. (2011) examined the effectiveness of personalized pre-performance music and imagery scripts in facilitating flow states and performance. Results indicated that the use of asynchronous music and imagery when combined showed facilitative effects on flow and perceived performance. Kuan et al. (2017) investigated the effect of unfamiliar relaxing and arousing music during imagery on physiological and subjectively perceived arousal of elite shooters. Results showed that music used during imagery helped to manipulate arousal in the required direction, and also that researcher-selected musical excerpts were at least as effective for arousal control as participant-selected excerpts. Despite the burgeoning literature on the impact of music on sports performance, there is still little research on the potential of music to enhance the effectiveness of imagery and its consequential influence on athletic performance. Given the widespread use of imagery training by athletes to enhance performance, the paucity of research on the potential of music to enhance imagery effectiveness represents a significant gap in knowledge.

Terry et al. (2014) identified more than 80 studies investigating effects of music on sports performance. However, most of those studies focused on exploiting the motivational qualities of music, and there is little research investigating the effect of music on performance via use of imagery training, or investigating the characteristics of music associated with changes to physiological arousal. It is important for the development of optimal imagery training programs that the impact of music is investigated when imagery training is temporally distanced from performance. In the small number of studies investigating effects of music and imagery in sport (e.g., Osborne, 1981; Dorney et al., 1992), performance was assessed directly after completing imagery with music, whereas athletes more typically use imagery techniques (with or without music) over a period of time in the build up to competitions (Terry and Karageorghis, 2011). A more ecologically valid approach would be for participants to practice imagery with music on a number of occasions over an extended period, with the impact on performance assessed at a later time.

In the present study, we investigated the effects of unfamiliar relaxing and arousing music to augment imagery training, on dart-throwing performance, physiological indicators of arousal, and self-reported competitive state anxiety. Dart-throwing was selected to evaluate performance because it is a self-paced, closed skill involving fine muscle control, and hence is not influenced by time pressure, the involvement of an opponent, or physical exertion. Such task characteristics minimize extraneous effects on arousal, making it easier to detect physiological changes in arousal due to the effects of music. Recently, Kuan et al. (2017) tested the psycho-physiological effects of music on skilled shooters during imagery. Results for GSR, PT, and electromyography measures showed that unfamiliar relaxing music (URM) was most relaxing and unfamiliar arousing music (UAM) was most arousing. Based on the findings of Kuan et al. (2017), it was hypothesized (a) that imagery training would have a positive effect on performance for all music conditions, (b) the performance effect for relaxing music would be greater than for arousing music and no music (NM), and (c) that relaxing music would be associated with lower physiological indicators of arousal, lower somatic and cognitive anxiety, and higher self-confidence than arousing music and NM.

## Materials and Methods

### Participants

Participants were 63 novice dart throwers (45 males, 18 females) aged 18–25 years (*M* = 20.21 years, *SD* = 3.20 years), recruited from students of sport and exercise science or physical education at a university in Melbourne, Australia. Participants had a minimum of one year’s experience playing sports that included Australian football, basketball, cricket, handball, netball, soccer, softball, swimming, taekwondo, tennis, track running, or volleyball. Four potential participants reported they had competitive dart-throwing experience and were excluded from the study. Females participated during their late luteal phase (i.e., around the 25th day of the menstrual cycle) to minimize psycho-physiological variations induced by hormonal changes. All participants reported their hearing as normal. Sample size was determined, based on a mixed design ANOVA, by using G^∗^Power 3.1.7. With an hypothesized effect size of 0.40, alpha of 0.05, power of 0.85, three groups, and two measurement occasions, the estimated sample size was 57. After adding the estimated drop-out rate of 10%, we required a total sample size of 63, with 21 participants in each condition.

### Measures and Materials

#### Demographic Information

Demographic information was collected about current participation in sport and/or physical activity; previous experience in sport, competition, and dart-throwing; sport imagery experience; and hearing ability.

#### Sport Imagery Ability Measure (SIAM; Watt et al., 2004)

The SIAM assesses imagery ability in sport by having athletes imagine four generic sport scenes for 60 s each; namely, the home venue, a successful competition, a slow start, and a training session. Following imagery of each scene, athletes respond to 12 items with reference to that scene by placing a cross on a 100-mm analog scale. The 12 imagery ability items cover five imagery dimensions (control, vividness, ease, speed of generation, duration), and six sensory modalities (kinaesthetic, tactile, visual, auditory, olfactory, and gustatory). In addition, one item assesses imagery of emotion experienced during each scene. The 12 subscales appear in different orders for each scene to minimize order effects. During its validation process, the SIAM demonstrated Cronbach’s alpha coefficients between 0.66 and 0.87 (Watt et al., 2004). We used the SIAM to ensure that participants had at least moderate imagery ability, to enable them to perform the dart-throwing imagery task. We asked participants to imagine their primary sport, rather than dart-throwing when completing the SIAM.

#### Imagery Script

A pre-recorded dart-throwing imagery script was developed and tested during a pilot phase of the study. The imagery script was checked for authenticity by two experienced darts players and minor changes implemented based on their feedback. The imagery script focused on the performance task, including feeling the weight of the dart, gripping the dart, looking at the bullseye, imaging the distance to the target and the height of the target from the floor, and releasing the dart accurately when ready. The imagery script also focused participants on the feeling in their arm muscles, the sensation of movement of the arm, and the need to keep their body still and stable as they threw. The script included instructions to picture successful performance with their darts hitting close to the bullseye, a strategy shown previously to build confidence leading to enhanced performance (Morris et al., 2005). Imagery training consisted of 12 sessions with an approximate duration of 9 min per session. Participants paced their own imagery and no instructions were provided about the frequency of imaging the dart throws.

#### Log of Imagery Sessions

Participants completed an adherence log to monitor the date, time, and duration of each imagery session, and added comments about their imagery experiences, such as how well they were able to concentrate and the emotions experienced. Completing the log provided an opportunity for participants to note changes in their experience over time and to comment on how strongly and vividly they experienced the music and imagery of dart-throwing. We established exclusion criteria, whereby participants with uncompleted logs and those who had missed more than two imagery sessions would be dropped from the main analysis. No participant met either criterion, indicating that all participants showed excellent adherence to the imagery intervention and completion of the log.

#### Music Conditions

Imagery training occurred under three music conditions using the same imagery script for all conditions. Unfamiliar music was used because it has been shown to generate similar levels of arousal to familiar music but minimizes the potential confounding effects of familiarity and past associations, which can lead to unpredictable effects on individual arousal level (Kuan et al., 2017). Music for the URM condition was taken from Frederick Delius’s Florida Suite: III Sunset “*Near The Plantation,*” which was selected from music excerpts confirmed in a preliminary study to be both relaxing and unfamiliar (Kuan et al., 2017). Music for the UAM condition was Edmond De Luca’s Conquerors of the Ages “*Attila The Hun,*” which was selected from music excerpts confirmed by Kuan et al. (2017) to be both arousing and unfamiliar. Participants in the NM control condition implemented imagery without music.

#### Physiological Signals

Physiological signal data were collected using the ProComp+ system and BioGraph software version 5.0 from *Thought Technologies*™ (Montreal, Canada), which assessed participants’ GSR, PT, and HR. To prepare physiological data for analysis, signal filtering was performed to remove noise and artifacts. The raw GSR signal was detrended using a piecewise linear regression model when sudden drift was noticed through visual inspection. No filtering was required for the PT and HR signals as the outputs were clean. When measuring physiological data, it is important to consider baseline differences. Thus, recordings were compared with baseline physiological data collected over a 5-min period of silent relaxation in a sitting position.

#### Galvanic Skin Response (GSR)

Two sensors were placed on the non-dominant hand of participants, comprising Ag/AgCl electrodes on the medial phalanges of the second and fourth digits. GSR is a linear inverse correlate to relaxation, and reflects emotional responses as well as cognitive activity (Lang, 1995). As sweat is produced in response to sympathetic nervous system activity, the capacity of the skin to conduct an electric current is enhanced and measured conductance is increased. Higher GSR scores reflect increased conductivity, indicating increased arousal.

#### Peripheral Temperature (PT)

A thermistor was positioned on the ventral side of the little finger of the non-dominant hand of participants (threshold = 0.0, scale – 0.25). PT is a measure of the temperature of the skin extremities, which varies according to the amount of blood perfusing to the skin and reflects sympathetic arousal of the autonomic nervous system. When individuals are sympathetically aroused, muscle contraction occurs. This causes vasoconstriction, which reduces blood flow to the skin, and results in a decrease in temperature (Kluger et al., 1985). Therefore, as arousal increases, PT decreases. Increases in PT reflect greater relaxation.

#### Heart Rate (HR)

Heart rate (HR) was measured using an EKG receiver (Thought Technology) coupled to a Polar HR transmitter worn around the chest. EKG receivers use wireless transmission and are more stable and less prone to movement artifacts than a blood volume pulse measure (BVP).

#### Competitive State Anxiety Inventory-2 Revised (CSAI-2R; Cox et al., 2003)

The CSAI-2R was used to assess somatic state anxiety (five items), cognitive state anxiety (seven items), and self-confidence (five items) prior to dart-throwing performance. Respondents rated their feelings on the 17-item instrument (e.g., *I feel jittery, I am concerned about losing*) using a 4-point Likert scale from 1 (*not at all*) to 4 (*very much so*). Subscale scores were derived by summing item scores in each subscale, dividing by the number of items, and multiplying by 10. Score range is 10–40 for each subscale. The factorial validity of the CSAI-2R was established by Cox et al. (2003), using confirmatory factor analysis on data from 331 athletes, which showed a good fit to the hypothesized measurement model (CFI = 0.95, NNFI = 0.94, RMSEA = 0.05) and Cronbach alpha coefficients showed sound internal consistency (somatic anxiety = 0.88, cognitive anxiety = 0.83, self-confidence = 0.85).

#### Dart-Throwing Performance

Dart-throwing is a self-paced, closed skill, involving fine-motor control, meaning that performance occurred in a stable, predictable environment in which performers could choose when to execute the task. As such, arousal level during dart-throwing was largely unaffected by the physical aspects of the task, which assisted in isolating the influence of external factors, such as music. A modified international-standard competition dartboard and five precision steel tip darts were used for the performance task. The dartboard had 10 concentric circles with diameters of 2, 4, 6, 8, 10, 12, 14, 16, 18, and 20 cm. Concentric circles were used in preference to an unmodified competition dartboard to minimize the effects of strategy. Participants scored 10 points for hitting the center circle (known as the bullseye), nine points for the next circle, down to one point for the outermost circle, and zero if the dart hit outside the outer circle or missed the dartboard. Pre- and post-intervention performances were based on 40 darts thrown from a distance of 237 cm with the bullseye at a height of 173 cm from the floor. Possible scores were in the range 0–400 points. Participants were instructed to stand behind the throwing line, aim for the bullseye, and throw when ready. Five practice trials preceded each performance test of 40 trials.

#### Short Interview

On completion of dart-throwing assessment at the post-intervention stage, six participants (two from each experimental condition) completed a short interview to describe their subjective experience of the imagery training. Participants shared their imagery experiences, the effects of the music, challenges they faced during the imagery training, and recommendations for use of music with imagery. Questions related to music were not asked of the two NM interviewees.

### Procedure

A pre-intervention – intervention – post-intervention design was used in this investigation, which ran over a 4-week period. First, participants provided written informed consent, completed a demographic information form, and were screened for imagery ability using the SIAM. Only participants who rated themselves as having moderate-to-high imagery ability participated in the study. Second, participants completed the CSAI-2R prior to the assessment of baseline dart-throwing performance. Third, participants were randomly assigned to three experimental conditions, termed URM, UAM, and NM, and undertook 12 sessions of imagery training while listening to their allocated music or NM over the 4-week period. Session 1 of the imagery training commenced 20 min after assessment of baseline dart-throwing performance.

For imagery sessions 2–11, participants listened to the 540 s imagery script on alternate days, using an MP3 player, and were prompted to do so via emails and telephone calls. Participants were instructed to follow the imagery script in a relaxed position while listening to the music and imaging the scene, and were asked to not conduct imagery training when tired. GSR, PT, and HR were monitored during sessions 1 and 12 of the imagery training, from 5 min before imagery commenced to 5 min after imagery ended. Fourth, after completing 12 sessions of imagery training, participants were retested on the dart-throwing performance task, having first completed the CSAI-2R. Finally, six participants (two in each condition) were selected to explore their subjective experiences using the interview protocol. All participants were debriefed and thanked for contributing to the study.

### Data Analyses

Quantitative data were collated for analysis using SPSS Version 23, and checked for missing data and outliers (none were found). Assumptions of the statistical procedures used were confirmed. Descriptive statistics were calculated for all study variables and between-condition differences in baseline data were assessed using one-way analysis of variance (ANOVA). Performance gain scores for the three music conditions were compared using one-way ANOVA, plus follow-up Tukey tests. Gain scores were calculated as the difference between post-intervention performance and pre-intervention performance scores. Physiological measures (GSR, PT, HR) monitored during Session 1 and Session 12 were plotted graphically over the 540-s imagery period (*t*_0_ to *t*_540_) and between-group differences were assessed using a two-way, mixed design ANOVA (condition × session) plus follow-up Tukey tests. Music condition (URM, UAM, NM) was the between-subject factor, and time (Session 1 vs. Session 12) was the within-subject factor. We also examined the interaction between music during imagery conditions and time for the physiological variables. Paired samples *t*-tests were used to assess changes in CSAI-2R scores (somatic anxiety, cognitive anxiety, self-confidence) from Session 1 to Session 12. In all cases where we made multiple comparisons, we applied the Bonferroni adjustment to significance levels to reduce the risk of Type 1 errors (Hair et al., 1998).

Qualitative data from the short interviews were summarized using inductive content analysis. Content analysis refers to investigators searching text for recurring words and themes (Patton, 2002). This procedure allows researchers to organize raw data (e.g., direct quotations from participants) into interpretable and meaningful themes and categories as the inquirers come to understand patterns that exist (Hanton and Jones, 1999). Inductive content analysis was used because the purpose of the short interviews was to explore the experience of the participants during imagery with music or NM. Inductive content analysis has been widely used by sport psychology researchers (e.g., Pink et al., 2015; Ludlam et al., 2016; Partridge and Knapp, 2016).

## Results

### Imagery Ability

Analysis of variance showed no significant between-condition differences on any SIAM subscale (see **Table 1**), indicating that participants in the three music conditions (URM, UAM, NM) did not differ in imagery ability at the start of the study. This result provides evidence that participants in the three music conditions were able to use imagery equally effectively as part of the intervention program.

**Table 1:** Sport imagery ability measure (SIAM) subscale scores for three music conditions (*N* = 63).

	URM		UAM		NM			
SIAM	*M*	*SD*	*M*	*SD*	*M*	*SD*	*F*_2,62_	*p*
Vividness	289.86	47.34	291.24	41.39	293.43	45.85	0.03	0.97
Control	287.24	51.58	279.67	42.31	282.62	50.52	0.13	0.88
Ease Generation	274.91	59.99	283.47	49.25	289.33	54.46	0.37	0.69
Speed Generation	280.57	53.71	285.47	42.63	288.62	47.41	0.15	0.86
Duration	274.14	58.92	276.24	49.36	282.47	59.07	0.13	0.88
Visual	292.38	46.22	291.81	37.60	294.95	49.41	0.03	0.97
Auditory	230.33	71.83	246.81	63.64	235.38	68.90	0.39	0.68
Kinaesthetic	252.57	53.42	235.28	53.78	253.76	53.92	0.78	0.46
Olfactory	168.33	74.16	121.38	60.64	144.00	61.53	2.68	0.08
Gustatory	156.24	85.44	114.86	69.53	133.09	71.32	1.57	0.22
Tactile	236.67	65.68	235.71	58.09	245.09	55.03	0.16	0.86
Emotion	247.05	61.46	261.95	47.99	247.95	52.39	0.43	0.66

*URM, unfamiliar relaxing music; UAM, unfamiliar arousing music; NM, no music.*

### Dart-Throwing Performance

**FIGURE 1:**
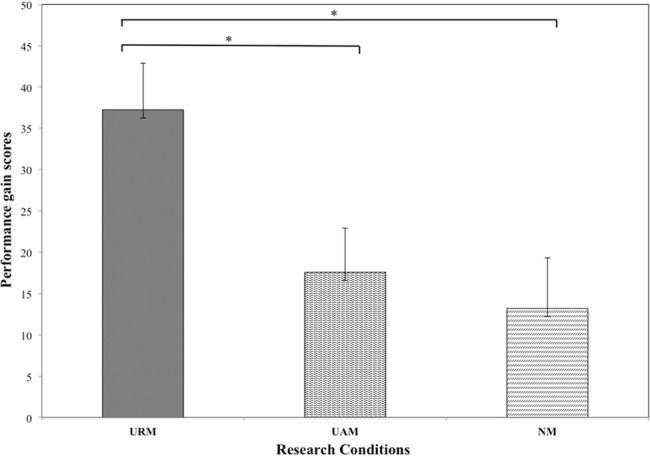
Dart-throwing performance gain scores for unfamiliar relaxing music (URM), unfamiliar arousing music (UAM) and no music (NM) conditions. ^∗^*p* < 0.05.

One-way ANOVA showed no significant difference in dart-throwing performance among the three music conditions at the pre-intervention stage (*F*_2,62_ = 1.15, *p* = 0.32, η^2^ = 0.04). Dart-throwing performance mean scores for URM, UAM, and NM, were 167.10 ± 37.97, 182.95 ± 49.46, and 186.05 ± 41.95, respectively, at the pre-intervention stage, and 204.34 ± 28.13, 200.52 ± 30.25, and 199.24 ± 32.64, respectively, at the post-intervention stage. The mean gain scores for dart-throwing performance from pre- to post-intervention are shown graphically in **Figure 1**. The URM group showed the greatest improvement in dart-throwing performance, followed by the UAM group, with the NM group showing the smallest gain.

Gain scores for the URM, UAM, and NM groups were 37.24 ± 5.66, 17.57 ± 5.30, and 13.19 ± 6.14, representing performance improvements of 22.3, 9.6, and 7.1%, respectively. ANOVA indicated a significant between-condition difference in gain scores (*F*_2,62_ = 5.03, *p* = 0.01, η^2^ = 0.14). Pairwise comparisons confirmed significant difference in gain scores between the URM group and the UAM group (*p* = 0.04) and NM group (*p* = 0.01), but no significant difference between the UAM and NM groups (*p* = 0.85).

### Physiological Indices

#### Galvanic Skin Response (GSR)

**FIGURE 2:**
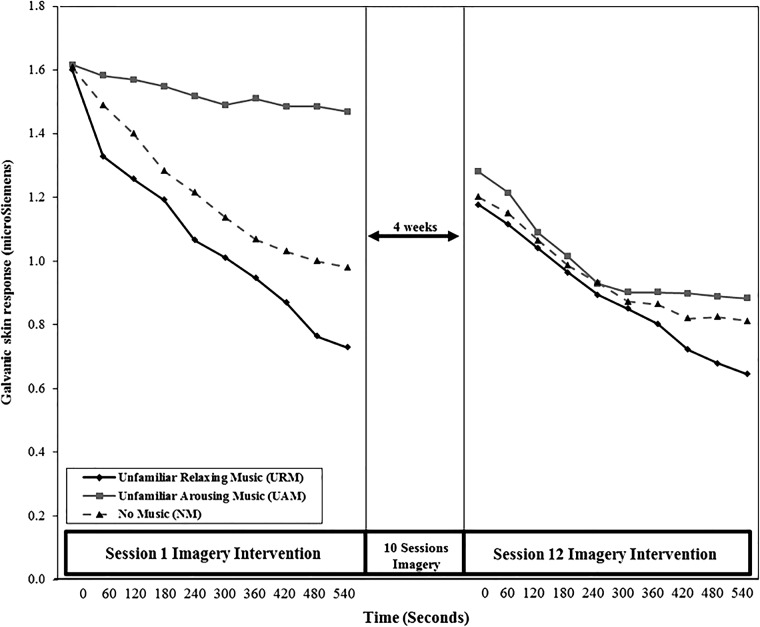
Mean galvanic skin response (GSR) from *t*_0_ to *t*_540_ across Sessions 1 and 12.

One-way ANOVA showed no significant difference among music conditions for GSR at baseline (*F*_2,62_ = 0.002; *p* = 0.99, η^2^ < 0.001). **Figure 2** shows mean values for GSR from *t*_0_ to *t*_540_ for each of the three experimental groups (UAM, URM, and NM) during Sessions 1 and 12. The line graphs indicate that the URM condition was the most relaxing for participants, with GSR decreasing monotonically over time throughout the imagery periods. GSR for the NM condition also decreased during Sessions 1 and 12, but the extent and the rate of decrease were less than for URM. The UAM condition was associated with the highest arousal levels during Sessions 1 and 12, although a decrease in GSR was evident from the start to end of each session, notably so in Session 12. GSR levels at the start of Session 12 were lower than at the start of Session 1 for all music conditions.

Comparing *t*_0_ values, a two-way, mixed design ANOVA (condition × time) showed a significant main effect of time (*F*_1,60_ = 6.17, *p* = 0.02, η^2^ = 0.09), indicating that GSR was lower across conditions at the start of Session 12 than at the start of Session 1. There was no main effect for condition (*F*_2,60_ = 0.05, *p* = 0.95, η^2^ = 0.002) and no significant condition × time interaction (*F*_2,60_ = 0.049, *p* = 0.95, η^2^ = 0.002). Comparing *t*_540_ values, a mixed design ANOVA (condition × time) showed a significant main effect of time (*F*_1,60_ = 10.05, *p* = 0.002, η^2^ = 0.14), indicating that GSR was lower across conditions at the end of Session 12 than at the end of Session 1. A significant main effect for condition was also evident (*F*_2,60_ = 4.68, *p* = 0.013, η^2^ = 0.14). *Post hoc* Tukey tests confirmed a significant difference between URM and UAM (*p* = 0.01), but no difference between URM and NM (*p* = 0.39) nor between UAM and NM (*p* = 0.20). A significant condition × time interaction was evident (*F*_2,60_ = 4.31, *p* = 0.02, η^2^ = 0.13). UAM was associated with significant reductions in GSR from end of Session 1 to end of Session 12, whereas GSR reduced only marginally for URM and NM over the same period (see **Figure 2**).

#### Peripheral Temperature (PT)

**FIGURE 3:**
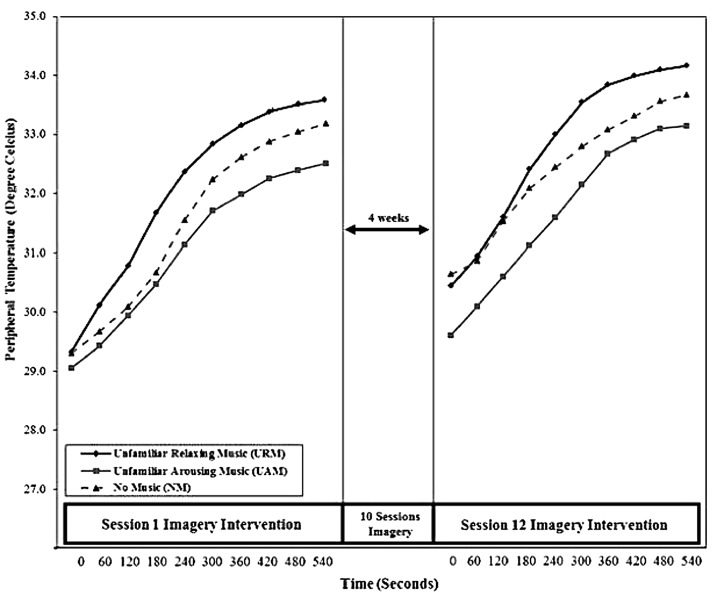
Mean peripheral temperature (PT) from *t*_0_ to *t*_540_ across Sessions 1 and 12.

Results of one-way ANOVA showed no significant difference among music conditions for PT at baseline (*F*_2,62_ = 0.03, *p* = 0.97, η^2^ < 0.001). **Figure 3** shows the mean values for PT from *t*_0_ to *t*_540_ for the three experimental groups during Sessions 1 and 12. There were no between-group differences at the start of Session 1, although the groups diverged as the session progressed, with URM showing the highest level of PT, indicating the lowest level of arousal. This pattern repeated during Session 12. However, this divergence was not of sufficient magnitude to produce statistically significant differences between conditions at the end of Sessions 1 and 12.

Comparing *t*_0_ values, a two-way, mixed design ANOVA (condition × time) showed no significant main effect of condition (*F*_2,60_ = 0.21, *p* = 0.81, η^2^ = 0.01) or time (*F*_1,60_ = 2.38, *p* = 0.13, η^2^ = 0.04), and no significant interaction between condition and time (*F*_2,60_ = 0.19, *p* = 0.83, η^2^ = 0.01). Comparing *t*_540_ values, a mixed design ANOVA (condition × time) similarly showed no significant main effect of condition (*F*_2,60_ = 1.32, *p* = 0.28, η^2^ = 0.04) or time (*F*_1,60_ = 0.08, *p* = 0.77, η^2^ = 0.001), and no significant interaction between condition and time (*F*_2,60_ = 0.16, *p* = 0.85, η^2^ = 0.01).

#### Heart Rate (HR)

**FIGURE 4:**
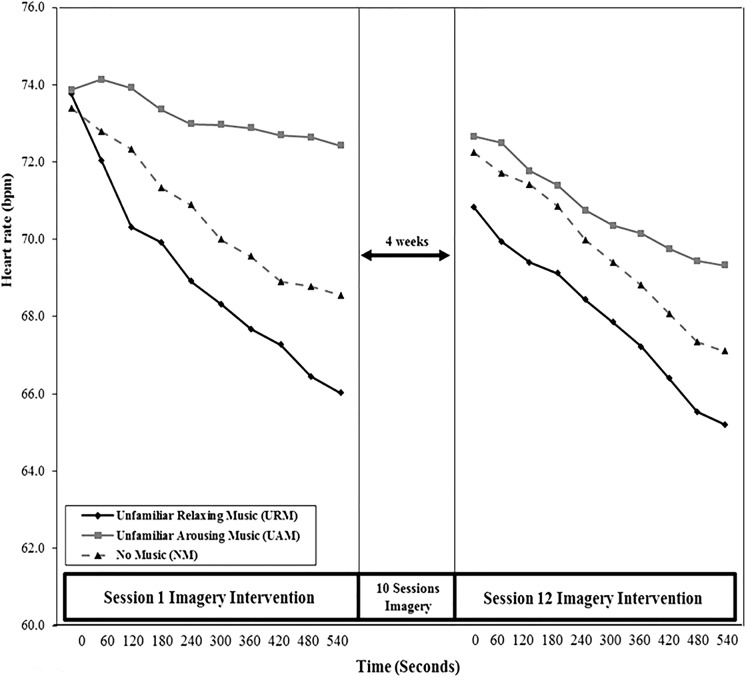
Mean heart rate (HR) from *t*_0_ to *t*_540_ across Sessions 1 and 12.

One-way ANOVA showed no significant difference among music conditions for HR at baseline (*F*_2,62_ = 0.02; *p* = 0.98, η^2^ < 0.001). **Figure 4** shows the HR means from *t*_0_ to *t*_540_ for the three experimental groups during Sessions 1 and 12. The URM group showed the largest reductions in HR across Sessions 1 and 12, with HR decreasing monotonically from the start to the end of both sessions. Comparing *t*_0_ values, a mixed design ANOVA (condition × time) showed no significant main effect of condition (*F*_2,60_ = 0.22, *p* = 0.80, η^2^ = 0.01) or time (*F*_1,60_ = 0.82, *p* = 0.37, η^2^= 0.01), and no significant interaction between condition and time (*F*_2,60_ = 0.27, *p* = 0.77, η^2^ = 0.01).

Comparing *t*_540_ values, a two-way, mixed design ANOVA (condition × time) showed a significant main effect of condition (*F*_2,60_ = 4.28, *p* = 0.02, η^2^ = 0.13). *Post hoc* Tukey tests confirmed that HR values were significantly lower for the URM group than the UAM group at the end of Sessions 1 and 12 (*p* = 0.01), but no significant differences were found between URM and NM (*p* = 0.44), or UAM and NM (*p* = 0.22). No significant main effect was found for time (*F*_1,60_ = 1.94, *p* = 0.17, η^2^ = 0.03), and no significant interaction between condition and time was found (*F*_2,60_ = 0.20, *p* = 0.82, η^2^ = 0.01).

### Competitive State Anxiety

Descriptive statistics for the CSAI-2R among participants in the three music conditions prior to the pre- and post-intervention assessments of dart-throwing performance are shown in **Table 2**. One-way ANOVA showed no significant difference among music conditions at baseline for somatic anxiety (*F*_2,62_ = 0.03, *p* = 0.97, η^2^ < 0.001), cognitive state anxiety (*F*_2,62_ = 0.20, *p* = 0.82, η^2^ = 0.01), self-confidence (*F*_2,62_ = 1.78, *p* = 0.18, η^2^ = 0.06). In paired-samples *t*-tests, we identified significant reductions in somatic and cognitive anxiety, and a significant increase in self-confidence, among the URM group (see **Table 2**). Cohen’s *d* values indicated that these changes from pre- to post-intervention represented very large effects. The UAM group reported a significant reduction in somatic anxiety, of moderate magnitude, but no significant changes in cognitive anxiety or self-confidence. The NM group reported a significant reduction in cognitive anxiety, of moderate-to-large magnitude, but no significant changes in somatic anxiety and self-confidence.

**Table 2:** Pre- and post-intervention scores for somatic anxiety, cognitive anxiety, and self-confidence (*N* = 63).

Source		Pre-intervention	Post-intervention	*t*_20_	*p*	*d*
**Somatic anxiety**					
URM	*M* (*SD*)	16.38 (3.53)	12.67 (2.76)	5.06	<0.001	1.10
UAM	*M* (*SD*)	16.05 (5.14)	14.38 (4.49)	2.34	0.03	0.51
NM	*M* (*SD*)	16.14 (4.93)	14.43 (4.49)	2.00	0.06	0.44
**Cognitive anxiety**					
URM	*M* (*SD*)	20.10 (5.78)	13.62 (4.63)	5.09	<0.001	1.11
UAM	*M* (*SD*)	20.86 (8.31)	17.90 (6.24)	2.05	0.06	0.45
NM	*M* (*SD*)	19.52 (6.13)	17.05 (6.68)	3.23	0.004	0.70
**Self-confidence**					
URM	*M* (*SD*)	26.29 (3.70)	33.33 (3.60)	6.97	<0.001	1.52
UAM	*M* (*SD*)	28.57 (5.63)	29.05 (7.23)	0.45	0.66	0.10
NM	*M* (*SD*)	28.86 (5.00)	30.76 (4.62)	2.12	0.06	0.46

*URM, unfamiliar relaxing music; UAM, unfamiliar arousing music; NM, no music.*

### Short Interview Data

All participants completed each of the 12 sessions of imagery training. Six participants reported on their overall imagery training experiences, and four participants commented on their experience of music during imagery. Participants also reported any challenges they faced during imagery training and recommendations for future sessions. Pseudonyms have been used to ensure participant anonymity. The participants, with corresponding research condition, were Christian (URM), Anna (URM), Andrew (UAM), Jackson (UAM), Matthew (NM), and Johnna (NM).

All six respondents indicated they had made a sustained effort throughout the imagery training and dart-throwing trials. Further, five of the six participants indicated that the intervention had improved their dart-throwing performance. Participants felt more relaxed, positive, confident, and experienced greater flow following the imagery intervention compared to during the pre-intervention dart-throwing trials, as exemplified by Christian’s comments:

The imagery showed some effects on the dart-throwing, I felt confident and great… it not only had helped my capacity to hold more images in my head, but it also affected my mood and the positive outcome… my body had less tension… I was able to imagine more flow state within the imagery…I felt in control of my task knowing what to react to and able to concentrate on my task.

Anna said, “After the imagery, I was relaxed… I felt my body was not tensed… I have more confidence to throw the darts on target and to improve my score… I am surprised this imagery could make a difference in my skills.” Andrew indicated he had experienced reduced anxiety and less concern about his performance: “I improved in my scoring… I felt less nervous compared to the initial competition… I was not worried… my confidence increased…” Matthew also indicated that, during the intervention phase, he had felt more relaxed, but it was after the third imagery training session that he began to note significant changes; “I was able to visualize the dart-throwing more efficiently after the third attempt… I began to feel relaxed… my body was not tense as before… I began to feel more focused and had more concentration… I felt good.” Similar responses were provided by Johnna: “Imagery works…my body was relaxed… I had complete concentration and focus… I was not worried about the scoring… I was not concerned about performing poorly.”

On the impact of music for imagery training, all four participants reported that they enjoyed having music as a facilitator to imagery training. For example, Anna expressed the view that during the imagery training, she felt more excitement and motivation, and the music had prompted her to remember the imagery: “I like the (relaxing) music … the rhythm was good… I am in a relaxed mood… it makes the imagery a more interesting, motivating and exciting experience… it helped me to remember the imagery during the post-performance.” Andrew also expressed excitement and motivation: “I found it an interesting and rather exciting experience… I experienced a motivating experience visualizing with high beat music… slowly I got used to the music and began to visualize my throwing more efficiently.” Jackson used the music to energize his imagery, “I used the music to ‘pump me up’ and make me perform better… it makes me more excited, want to do it better, getting more aroused, but then ‘turn them off’ to focusing on throwing the dart… I think it is a fun experience.”

As for the challenges faced, two participants reported difficulties trying to concentrate, occasionally feeling tired, and finding suitable times to complete the imagery sessions. For example, Matthew expressed the following thoughts:

I nearly missed one or two sessions due to tiredness… I have assignments and a lecture in the morning…. it was rather hard to concentrate if I was tired… However, the follow-up on contacting me and completion of the logbook did help me to remember to do my training.

Johnna reported similar concerns: “I was distracted twice… waking up too early made me de-motivated and tired… it was hard to manage my time for the imagery.”

Finally, participants suggested recommendations, including longer imagery periods (Christian, Matthew), shorter imagery periods (Andrew), weekly follow-ups (Matthew), having a performance test between imagery sessions (Anna), measuring HR in every session (Matthew), adding a breathing exercise before imagery (Johnna), using their own earphones (Jackson), and increasing the frequency of imagery sessions (Anna, Matthew). Matthew said, “I think it should be more, as 12 sessions is not enough for me. I suggest 20 sessions will be ideal…”

## Discussion

We investigated effects of unfamiliar relaxing and arousing music during imagery training on subsequent dart-throwing performance, physiological arousal indices, and competitive state anxiety. Imagery ability was confirmed as being sufficiently well developed and equal across groups, prior to participants completing 12 sessions of imagery training accompanied by one of three music conditions (URM, UAM, NM) over a 4-week period. Dart-throwing performance was assessed before and after the intervention. Differences in mean performance scores among the music conditions at the pre-intervention stage were not statistically significant. Performance gain scores among the music conditions were evaluated rather than comparing specific pre- and post-intervention scores, as recommended by Huck and McLean (1975), as a statistical strategy to minimize the effect of pre-intervention differences between groups. As hypothesized, imagery training had a positive effect on performance for all music conditions and URM was associated with the highest performance gain scores. Participants who listened to URM improved significantly more (22.3%) than those in the UAM (9.6%) and NM (7.1%) groups.

Improvement by the NM group can be attributed to the combined benefits of the imagery intervention and the practice effect of completing the dart-throwing task twice. The additional improvement by the UAM group can be attributed to the augmentation of imagery with arousing music, whereas the much greater improvement by the URM group highlights the importance of using relaxing rather than arousing music to accompany imagery in order to maximize the performance benefit in fine motor skills, such as dart-throwing. Dorney et al. (1992) reported that music did not enhance the benefits of imagery on dart-throwing performance, although the methodological limitations of that study have been noted (Karageorghis and Terry, 1997). Other studies are more aligned with our findings. A triangulation interview study on NCAA Division 1 collegiate athletes concluded that music is an important facilitator of imagery (Sorenson et al., 2008), becoming an essential element in athletes’ pre-performance imagery routines. Athletes reported that music enabled them to focus on their imagery routine, block out distractions, and reduce anxiety. Further, in his seminal work on visuo-motor behavior rehearsal, Suinn (1976) proposed the benefits of preceding imagery rehearsal with a relaxation exercise. Our results support Suinn’s proposition by demonstrating that performance benefits were greater when imagery was used in conjunction with relaxing music.

Results from the physiological measures indicated consistent changes in GSR, PT, and HR that reflected greater reductions in arousal for the URM group than for either the UAM or NM groups. Visual inspection of **Figures 2**–**4** shows consistent patterns of responses, wherein the three groups commenced the imagery sessions at similar levels of arousal but steadily diverged during the 9 min that the imagery sessions lasted. Relaxing music, as hypothesized, was associated with the greatest reductions in arousal, arousing music was associated with the smallest reductions in arousal, with the NM group falling in between the two music conditions. Although these trends are unsurprising, it is noteworthy that the combination of imagery and arousing music produced a clear relaxation effect, rather than increasing arousal, during the imagery training sessions. It is also noteworthy that imagery produced a relaxation effect over the course of the sessions even in the absence of music. A practice effect was apparent, in that all three groups commenced and finished Session 12 at lower levels of arousal than the equivalent time in Session 1. The significant interaction effect between condition and time for GSR may indicate habituation to the UAM by the UAM group. In essence, the unfamiliar may have become familiar over the course of 12 imagery sessions, reflected in arousal declining only marginally during Session 1 due to the novelty of the arousing music, but declining significantly during Session 12 as the music became more familiar and its arousing qualities declined. This effect is more apparent for GSR measures compared to PT and HR, suggesting that among the three psycho-physiological indices used, GSR is the most sensitive indicator of autonomic nervous system activity. Kuan et al. (2017) reported similar results for GSR compared to PT and HR.

Self-report measures were included in the present study to assist interpretation of physiological changes in arousal level. Greater improvements in CSAI-2R scores were evident for the URM group than the other two groups from the pre- to post-intervention dart-throwing assessments. These results provide support for the proposition that reductions in physiological arousal were associated with relaxation rather than boredom or drowsiness. It is likely that both the imagery process and the music contributed to the observed changes in state anxiety and self-confidence scores. According to Morris et al. (2005), numerous studies in sport have shown that imagery training can decrease anxiety and increase self-confidence, often resulting in enhanced performance. For example, Hanrahan and Vergeer (2001) demonstrated that an imagery intervention provided for modern dancers reduced state anxiety and increased self-confidence, as well as helping to clarify goals and focus attention.

Most imagery studies that recorded state anxiety and self-confidence scores did so because the imagery intervention was designed specifically to reduce anxiety or enhance confidence. Although this was not the primary focus of our study, participants were encouraged to imagine successful performance outcomes, a strategy previously shown to enhance self-confidence (e.g., Wesch et al., 2016). The use of relaxing music during imagery in the present study may have played an important role in reducing state anxiety. For example, Elliott et al. (2012) provided support for the application of relaxing music in reducing competitive state anxiety and Seaward (2002) recommended that listening to relaxing music for 1 h a day could significantly reduce anxiety.

Researchers have reported that increases in self-confidence tend to accompany reductions in state anxiety (Woodman and Hardy, 2003). Our results supported the proposal that listening to relaxing music during imagery was effective in decreasing competitive state anxiety and increasing self-confidence for fine motor skill performance. The subjective experiences of participants derived from the interviews at the end of the study indicated that they benefited from imagery regardless of research condition. Imagery was associated with improvements in performance, enhanced relaxation, and a more positive mindset. In addition, all four interview participants who completed imagery with music reported that the music created more motivation, fun, and excitement, which motivated them to perform the imagery training.

One limitation of the present study was that we provided no instructions to participants about the number of times they should imagine throwing a dart at the target during each session. Instead, participants paced their own imagery. Hence, there was inconsistency in number of throws imagined, which may have influenced performance. However, we found no study in the literature that had assessed the impact of number of imagery repetitions on performance. The evidence we gleaned from observing participants and inviting their feedback in logs and interviews suggested there were no systematic differences in number of imagery repetitions between the three experimental conditions. Nevertheless, we recommend that the number of imagery repetitions is controlled in future studies in this area of investigation.

Given that we interviewed only two participants from each music condition, their responses have limited generalizability and we recommend that additional qualitative investigations be conducted to explore the effects of music on imagery more fully. Differences in dart-throwing performance among the three music conditions at the pre-intervention stage are also noted. Although these differences were not significant and use of gain score analysis ameliorated their potential effects, we recommend that future studies allocate participants to music conditions using matched pre-intervention performance scores.

### Future Research

Our results raised a number of considerations that warrant further investigation. For example, some researchers (e.g., Karageorghis and Priest, 2012) have emphasized the benefits of music for sport performance as part of pre-competition routines, and Terry (2004) provided specific examples of how elite athletes have used music within pre-competition routines to regulate mood and arousal. However, we found no studies that examined effects of music presented in contexts temporally removed from performance. Given that some sports prohibit athletes from using any form of music player in the competition venue, it may be difficult for performers to use music as part of their pre-competition routines during events. Therefore, further investigations of the extent to which a program of imagery with music completed well in advance of competition might benefit subsequent performance, would be worthwhile.

One benefit of examining the impact of imagery on novices is that they have greater scope for improvement than more experienced performers. Some researchers have reported that novices may benefit more than elite performers from listening to music in the context of sporting performance (e.g., Karageorghis and Priest, 2012) whereas others have argued that imagery is more effective with skilled performers than novices (Blair et al., 1993; Morris et al., 2005). A challenge for future research would be to clarify whether imagery conducted in combination with music provides performance benefits for highly skilled performers and therefore we recommend that the present study is replicated among elite athletes using context-specific performance tasks rather than dart-throwing. In particular, it would be worthwhile to investigate the impact of imagery with different music conditions on the performance of gross motor skills of a more dynamic nature rather than the fine motor skill used in the present study.

## Conclusion

Results provided strong support for using imagery training accompanied by URM to enhance performance of a fine motor skill. Further examination of the effects of relaxing and arousing music on other sports tasks and among participant groups of varying performance levels, is recommended.

## Ethics Statement

The study was carried out in accordance with the recommendations from the Victoria University Human Research Ethics Committee with written informed consent from all participants. All participants gave written informed consent in accordance with the Declaration of Helsinki. The protocol was approved by the Victoria University Human Research Ethics Committee.

## Author Contributions

All authors listed have made a substantial, direct and intellectual contribution to the work, and approved it for publication.

## Conflict of Interest Statement

The authors declare that the research was conducted in the absence of any commercial or financial relationships that could be construed as a potential conflict of interest.

## References

[B1] Blair A. Hall C. Leyshon G. (1993). Imagery effects on the performance of skilled and novice soccer players. *J. Sports Sci.* 11 95–101. 10.1080/02640419308729971 8497020

[B2] Bood R. J. Nijssen M. van der Kamp J. Roerdink M. (2013). The power of auditory-motor synchronization in sports: enhancing running performance by coupling cadence with the right beats. *PLOS ONE* 8:e70758. 10.1371/journal.pone.0070758 23951000 PMC3737354

[B3] Boutcher S. H. Trenske M. (1990). The effects of sensory deprivation and music on perceived exertion and affect during exercise. *J. Sport Exerc. Psychol.* 12 167–176. 10.1123/jsep.12.2.167

[B4] Burns J. L. Labbé E. Williams K. McCall J. (1999). Perceived and physiological indicators of relaxation: as different as Mozart and Alice in Chains. *Appl. Psychophysiol. Biofeedback* 24 197–202. 10.1023/A:1023488614364 10652638

[B5] Collins D. Hale B. D. (1997). Getting closer...but still no cigar! Comments on Bakker, Boschker and Chung (1996). *J. Sport Exerc. Psychol.* 19 207–212. 10.1123/jsep.19.2.207

[B6] Cox R. H. Martens M. P. Russell W. D. (2003). Measuring anxiety in athletics: the revised Competitive State Anxiety Inventory-2 and sport performance: a meta-analysis. *J. Sport Exerc. Psychol.* 25 44–65. 10.1123/jsep.25.4.519

[B7] Dorney L. Goh E. K. M. Lee C. (1992). The impact of music and imagery on physical performance and arousal: studies of coordination and endurance. *J. Sport Behav.* 15 21–31.

[B8] Elliott D. Polman R. Taylor J. (2012). The effects of relaxing music for anxiety control on competitive sport anxiety. *Eur. J. Sport Sci.* 14 (Suppl. 1), S296–S301. 10.1080/17461391.2012.693952 24444221

[B9] Grocke D. Wigram T. (2007). Receptive methods in music therapy: techniques and clinical applications for music therapy clinicians, educators and students. *Music Ther. Perspect.* 25 127–129. 10.1093/mtp/25.2.127

[B10] Hair J. E. Jr. Anderson R. E. Tatham R. L. Black W. C. (1998). *Multivariate Data Analysis*, 5th Edn. Englewood Cliffs, NJ: Prentice-Hall.

[B11] Hall C. R. (2001). “Imagery in sport and exercise,” in *Handbook of Research on Sport Psychology*, 2nd Edn, eds Singer R. N. Hausenblas H. A. Janelle C. M. (New York, NY: John Wiley), 529–549.

[B12] Hall C. R. Mack D. E. Paivio A. Hausenblas H. A. (1998). Imagery use by athletes: development of the sport imagery questionnaire. *Int. J. Sport Psychol.* 29 73–89.

[B13] Hanrahan C. Vergeer I. (2001). Multiple uses of mental imagery by professional modern dancers. *Imagin. Cogn. Pers.* 20 231–255. 10.2190/RLBE-XQK9-C65F-X05B

[B14] Hanton S. Jones G. (1999). The acquisition and development of cognitive skills and strategies: making the butterflies fly in formation. *Sport Psychol.* 13 1–21. 10.1123/tsp.13.1.1

[B15] Huck S. W. McLean R. A. (1975). Using a repeated measures ANOVA to analyze the data from a pretest-posttest design: a potentially confusing task. *Psychol. Bull.* 82 511–518. 10.1037/h0076767

[B16] Hutchinson J. C. Karageorghis C. I. (2013). Moderating influence of dominant attentional style and exercise intensity on psychological and psychophysical responses to asynchronous music. *J. Sport Exerc. Psychol.* 35 625–643. 10.1123/jsep.35.6.625 24334323

[B17] Karageorghis C. I. Lee J. (2001). “Effects of asynchronous music and imagery on an isometric endurance task,” in *Proceedings of the World Congress of Sport Psychology: International Society of Sport Psychology*, Vol. 4 (Skiathos: ISSP), 37–39.

[B18] Karageorghis C. I. Priest D. L. (2012). Music in the exercise domain: a review and synthesis (part I). *Int. Rev. Sport Exerc. Psychol.* 5 67–84. 10.1080/1750984X.2011.631027 22577473 PMC3339577

[B19] Karageorghis C. I. Terry P. C. (1997). The psychophysical effects of music in sport and exercise: a review. *J. Sport Behav.* 20 54–68. 22512537

[B20] Kluger M. A. Jammer L. D. Tursky B. (1985). Comparison of the effectiveness of biofeedback and relaxation training on hand warming. *Psychophysiology* 22 162–166. 10.1111/j.1469-8986.1985.tb01580.x 3887452

[B21] Kuan G. Morris T. Terry P. (2017). Effects of music on arousal during imagery in elite shooters: a pilot study. *PLOS ONE* 12:e0175022. 10.1371/journal.pone.0175022 28414741 PMC5393549

[B22] Lang P. J. (1995). The emotion probe. *Am. Psychol.* 50 372–385. 10.1037/0003-066X.50.5.372 7762889

[B23] Ludlam K. E. Butt J. Bawden M. Lindsay P. Maynard I. W. (2016). A strengths-based consultancy approach in elite sport: exploring super-strengths. *J. Appl. Sport Psychol.* 28 216–233. 10.1080/10413200.2015.1105881

[B24] Lundqvist L. O. Carlsson F. Hilmersson P. Juslin P. N. (2009). Emotional responses to music: experience, expression, and physiology. *Psychol. Music* 37 61–90. 10.1177/0305735607086048

[B25] Morris T. Spittle M. Watt A. P. (2005). *Imagery in Sport.* Champaign, IL: Human Kinetics.

[B26] Osborne J. W. (1981). The mapping of thoughts, emotions, sensations, and images as responses to music. *J. Ment. Imag.* 5 133–136.

[B27] Pain M. A. Harwood C. Anderson R. (2011). Pre-competition imagery and music: the impact on flow and performance in competitive soccer. *Sport Psychol.* 25 212–232. 10.1123/tsp.25.2.212

[B28] Partridge J. A. Knapp B. A. (2016). Mean girls: adolescent female athletes and peer conflict in sport. *J. Appl. Sport Psychol.* 28 113–127. 10.1080/10413200.2015.1076088

[B29] Patton M. Q. (2002). *Qualitative Evaluation and Research Methods*, 3rd Edn. Thousand Oaks, CA: Sage Publications.

[B30] Perry C. Morris T. (1995). “Mental imagery in sport,” in *Sport Psychology: Theory, Applications and Issues*, eds Morris T. Summers J. (Sydney, NSW: John Wiley), 339–385.

[B31] Pink M. Saunders J. Stynes J. (2015). Reconciling the maintenance of on-field success with off-field player development: a case study of a club culture within the Australian Football League. *Psychol. Sport Exerc.* 21 98–108. 10.1016/j.psychsport.2014.11.009

[B32] Priest D.-L. Karageorghis C. I. Sharp N. C. (2004). The characteristics and effects of motivational music in exercise settings: the possible influence of gender, age, frequency of attendance, and time of attendance. *J. Sports Med. Phys. Fitness* 44 77–86. 15181394

[B33] Salimpoor V. N. Benovoy M. Larcher K. Dagher A. Zatorre R. J. (2011). Anatomically distinct dopamine release during anticipation and experience of peak emotion of music. *Nat. Neurosci.* 14 257–262. 10.1038/nn.2726 21217764

[B34] Salimpoor V. N. Benovoy M. Longo G. Cooperstock J. R. Zatorre R. J. (2009). The rewarding aspects of music listening are related to degree of emotional arousal. *PLOS ONE* 4:e7487. 10.1371/journal.pone.0007487 19834599 PMC2759002

[B35] Seaward B. L. (2002). *Managing Stress: Principles and Strategies for Health and Wellbeing*, 3rd Edn. Sudbury, MA: Jones and Bartlett.

[B36] Sorenson L. Czech D. R. Gonzales S. Klein J. Lachowetz T. (2008). Listen up: the experience of music in sport: a phenomenological investigation. *Athl. Insight* 11 1–18.

[B37] Strachan L. Munroe-Chandler K. (2006). Using imagery to predict self-confidence and anxiety in young elite athletes. *J. Imag. Res. Sport Phys. Act.* 3 1–19. 10.2202/1932-0191.1004

[B38] Suinn R. M. (1976). “Visual motor behaviour rehearsal for adaptive behaviour,” in *In Counseling Methods*, eds Krumboltz J. Thoresen C. (New York, NY: Holt, Rinehart & Winston), 360–366.

[B39] Terry P. C. (2004). “Mood and emotions in sport,” in *Sport Psychology: Theory, Applications and Issues*, 2nd Edn, eds Morris T. Summers J. (Brisbane, QLD: John Wiley), 48–73.

[B40] Terry P. C. Curran M. L. Karageorghis C. I. (2014). “Does music really make a difference? Meta-analytic review of a century of research,” in *Proceedings of the 28th International Congress of Applied Psychology*, Paris.

[B41] Terry P. C. Karageorghis C. I. (2011). “Music in sport and exercise,” in *The New Sport and Exercise Psychology Companion*, eds Morris T. Terry P. C. (Morgantown, WV: Fitness Information Technology), 359–380.

[B42] Terry P. C. Karageorghis C. I. Mecozzi Saha A. D’Auria S. (2012). Effects of synchronous music on treadmill running among elite triathletes. *J. Sci. Med. Sport* 15 52–57. 10.1016/j.jsams.2011.06.003 21803652

[B43] Watt A. P. Morris T. Andersen M. B. (2004). Issues of reliability and factor structure of sport imagery ability measures. *J. Ment. Imag.* 28 112–125.

[B44] Wesch N. Callow N. Hall C. Pope J. P. (2016). Imagery and self-efficacy in the injury context. *Psychol. Sport Exerc.* 24 72–81. 10.1016/j.psychsport.2015.12.007

[B45] Woodman T. Hardy L. (2003). The relative impact of cognitive anxiety and self-confidence upon sport performance: a meta-analysis. *J. Sport Sci.* 21 443–457. 10.1080/0264041031000101809 12846532

